# Indigenous Resilience in Australia: A Scoping Review Using a Reflective Decolonizing Collective Dialogue

**DOI:** 10.3389/fpubh.2021.630601

**Published:** 2021-03-31

**Authors:** Kim Usher, Debra Jackson, Roz Walker, Joanne Durkin, Reakeeta Smallwood, Melanie Robinson, Uncle Neville Sampson, Isabelle Adams, Cheryl Porter, Rhonda Marriott

**Affiliations:** ^1^School of Health, University of New England, Armidale, NSW, Australia; ^2^Ngangk Yira Research Centre for Aboriginal Health and Social Equity, Faculty of Health Sciences, Murdoch University, Perth, WA, Australia; ^3^Susan Wakil School of Nursing University of Sydney, Sydney, NSW, Australia; ^4^School of Indigenous Studies, University of Western Australia, Perth, WA, Australia; ^5^Faculty of Health, University of Technology Sydney, Sydney, NSW, Australia; ^6^Western Australian, Department of Health, Perth, WA, Australia; ^7^Telethon Kids Institute, University of Western Australia, Perth, WA, Australia; ^8^New South Wales Department of Health, North Sydney, NSW, Australia

**Keywords:** Aboriginal, indigenous peoples, Torres Strait Islander, resilience, decolonization, scoping review, literature review, Australia

## Abstract

Contemporary definitions and understandings of resilience refer to an individual's positive adaptation to the experience of adversity. One of the challenges of this extant body of work is that the central concept of resilience is rarely questioned. Current understandings of these concepts, largely framed in Western understandings, are unquestioningly accepted, reframed for, yet not by, Indigenous peoples, and then are unchallenged when imposed on Indigenous peoples. A scoping review was conducted and reported in line with the PRISMA-ScR guidelines. The review involved the participation of local Aboriginal Research Cultural Advisory Groups who participated and approved the analysis of the findings and collaborated on the design and writing of the paper. Eight publications drew on Aboriginal constructs of resilience in examining the effectiveness of programs, processes, and practices to promote individual and/or collective resilience and well-being. Most studies emphasized the need for strategies to strengthen individual or community connection to culture to foster resilience. Six studies used culturally validated strength-based tools to measure resilience, while two relied on Western constructs. This review reveals both the distinctive colonial characteristics of adversity experienced by Aboriginal people and the range of coping strategies and protective resources that support the development of resilience within different Aboriginal communities in diverse research sites across Australia. Importantly, many studies confirm adversity is linked to the enduring legacies of colonization, continuous and cumulative transgenerational grief and loss, structural inequities, racism, and discrimination. These external factors of adversity are unique to Aboriginal populations, as are the protective factors that entail strengthening connection to culture (including language reclamation), community, ancestry and land (including management and economic development) which contribute to individual and collective resilience. These findings suggest that Aboriginal community resilience is strengthened through the collective experience of adversity, such as transgenerational grief and loss, and the resulting support structures and shared resources that are developed and maintained through cultural practices to strengthen the bonds and mutual reciprocity to participate in transformative strategies to address adversity. This review highlights that strategies such as building on community strengths, capacities, and resources is critical when strengthening resilience within Indigenous communities across Australia.

## Introduction

Contemporary definitions of resilience refer to an individual's positive adaptation to the experience of adversity. Indigenous resilience is a complex phenomenon which relies on the positive adaptation of the individual, the community and the environment to adversity. Indigenous Peoples of Australia, like most other Indigenous populations globally, experience higher levels of adversity than non-indigenous people with a greatly disproportionate burden of disease, disability, premature mortality, and pervasive health inequalities over many decades. For example, Indigenous Peoples are more likely to smoke, engage in unsafe alcohol use, experience psychological distress and suicide, exercise less and have greater risk of circulatory and cardio-vascular problems (1). Challenges such as these are underpinned by persistent and continued racism, and various entrenched historical, social, and economic determinants. These factors together constitute a complex, multifaceted and pervasive colonial legacy that includes severe economic and health inequity (2).

Clearly, given these complex and pervasive challenges, further interrogation into understandings of Indigenous resilience is needed. There are several published reviews that examine resilience in the context of Indigenous peoples, many focussing on young people [see for example: (3, 4)]. With the exception of Jongen et al. (5) these tend to be structured around extant Western definitions of resilience. One of the challenges of this body of work as it stands is that the central concept of resilience is rarely questioned, and neither are other closely related concepts such as adversity and risk. Rather, the current understandings of these concepts, largely framed in Western understandings, are unquestioningly accepted, and imposed on Indigenous Peoples. Thus, these reviews are limited by a form of cultural imperialism that we argue has simply promulgated Western understandings and conceptions of resilience. In fact, the tendency to use non-indigenous measures to define, measure and quantify resilience in Indigenous Peoples has previously been noted in the literature (5).

Much work is conducted and interpreted through a non-indigenous worldview—few authors are identifiable as Indigenous, and even fewer indicate any authentic engagement with Indigenous individuals and communities in developing protocols and interpreting results. Furthermore, much that is “known” about Indigenous resilience is based on research data drawn from international Indigenous populations. While this work has value and is important, we argue that findings and insights arising from studies conducted internationally cannot be assumed to be fully relevant to Indigenous peoples in Australia. It is evident that although some aspects of resilience for Indigenous peoples may resonant globally; there may be others that are culturally and context specific to Australia which we wanted to capture. Initially, we conducted a preliminary review of articles and upon reflection, realized we had in fact perpetuated the exact same renditions of resilience that had gone before us. Chambers et al. (6) described the tension they experienced when trying to apply the inclusion/exclusion criteria to their review. Similarly, we ended up with literature that told the same story we had read previously on numerous occasions.

As our intent was to provide a review of resilience from an Indigenous perspective, we worked closely as a team and with local Aboriginal Research Cultural Advisory Groups to decide how to adopt a decolonizing approach to conduct the review. In particular, it was important that we were authentic in adopting a different lens to ensure we challenged Western understandings (7) and as a result, we altered our search terms to more appropriately reflect a decolonizing approach to the literature. Our review confirmed that there is very little research that has focused on Indigenous perspectives of resilience. While there is substantial research focused on the strengths and protective factors associated with cultural and well-being outcomes, few studies incorporate the concept of resilience as an explicit focus relevant to Indigenous peoples.

## Indigenous Concepts of Resilience

In this current review, we are aiming to more meaningfully and effectively incorporate Indigenous ways of knowing, being and doing to produce authentic insights and understandings of resilience that are more culturally inclusive than has previously been achieved. We have active Indigenous involvement through having six Indigenous persons on the author team (RM, RS, MR, IA, CP, NS), and through a process of engagement with local Indigenous communities and Elders in two geographically disparate sites. We are also focussing on data derived from research solely conducted within Australia, with and by Indigenous People. Through these strategies, we aim to provide a more nuanced understanding of what constitutes resilience from the perspectives of Australia's Indigenous Peoples. A preliminary search of PROSPERO, MEDLINE, the Cochrane Database of Systematic Reviews and the *JBI Database of Systematic Reviews and Implementation Reports* was conducted and no current or underway systematic reviews on the topic were identified.

## Aim

The aim of this scoping review was to incorporate Indigenous ways of knowing, being, and doing to produce authentic insights and more culturally inclusive understandings of resilience than has previously been achieved. This review explores the existing literature pertinent to Indigenous Australian Resilience. Key characteristics and key concepts of Indigenous Australian Resilience are explored.

Specific aims of this review were to:

1) Examine the scope of information in Australia reflecting Indigenous people's perspectives on, and understandings of, resilience.
2) Clarify key concepts and definitions of resilience from the perspectives of Indigenous peoples in Australia.
3) Examine key characteristics or factors of resilience from the perspectives of Indigenous peoples in Australia.
4) Examine how research is conducted on Indigenous resilience in Australia.

## Methodology

This scoping review was undertaken from an Indigenist position to articulate an alternative position to Indigenous resilience from the accepted dominant Western view. As Chambers et al. (6) remind us, health research has been criticized because of its negative social constructions of Indigenous Peoples that serve to perpetuate populations as vulnerable while reaffirming racialized stereotypes. We adopted the decolonizing methodologies and methods of Smith (7) and Kovach (8) to interrogate the evidence from the perspective of Indigenous Peoples. We limited the review to Australian evidence to provide a context specific Indigenist perspective, and reviewed the underpinning theoretical constructions of, and tools used to measure resilience, as well as concepts of adversity and the critical role of culture and context in resilience. Similar to the review of Chambers et al. (6), we incorporated critical reflection to the review process. This helped to ground the work in Indigenous worldviews, while the reflective processes incorporated ancestral or cultural wisdom (9), and facilitated deep listening and engagement (8).

We are a diverse team that includes two Indigenous scholars (RM, RS) one Indigenous health professional (MR), three Indigenous community members (NS, CP, IA) and four non-indigenous scholars with extensive experience in Indigenous health and well-being research. All members respect and uphold the need for alternative epistemologies that question the dominant knowledge paradigm and adopt a critically reflexive position in order to privilege Indigenous ways of knowing, being and doing. Our positioning as a team created an opportunity to engage with the literature through a research agenda that aligned our collaborative understandings with Indigenous epistemologies and this is highlighted throughout our analysis of the literature (7).

## Methods

A scoping review (SR) was selected as SR are useful when clarification around a concept or theory is required (10). The SR was conducted in accordance with the Joanna Briggs Institute methodology for scoping reviews (11) and reported in line with the PRISMA-ScR reporting guidelines (12). The completed checklist is included as Supplementary File 1. The review involved the participation of a local Aboriginal Research Cultural Advisory Group in each research site (Western Australia, New South Wales) who advised on the search strategy, participated in approved the analysis of the findings, and collaborated on the design of the paper.

### Inclusion and Exclusion Criteria

Studies were included if they were peer reviewed, primary research focussing on Aboriginal and Torres Strait Islander perspectives and understanding of resilience in Australia. The inclusion and exclusion criteria for selecting articles was established (Table 1).

**Table 1:** Inclusion and exclusion criteria for selecting articles.

**Inclusion criteria**	**Exclusion criteria**
• Peer-reviewed, primary research • Focusing on Aboriginal and Torres Strait Islander (populations) perspectives and understanding of resilience (concept) in Australia (context) • Published in English • Published between January 1990 and June 2020	• No engagement or collaboration with population; research on, not with Australian Aboriginal and Torres Strait Islanders • No conceptualization of resilience from an Indigenous perspective i.e., Western conceptualization of resilience applied

### Search Strategy

The literature search for this scoping review was carried out between June and August 4. The search adopted a three-step approach as outlined in the JBI Manual for scoping reviews (11). This involved searching relevant databases to analyze search terms and text words captured in the databases; completing a second search with the revised search terms across all databases, and searching of the reference lists of identified studies (13). Initial searches were conducted in CINAHL Complete and Informit Indigenous Collection. The initial search strategy was developed in consultation with a University health librarian. The text words contained in the titles and abstracts of relevant articles, and the index terms used to describe the articles were used to develop a full search strategy. These terms were discussed with the Aboriginal Research Cultural Advisory Group members partnering on this project and it was agreed to add 11 additional terms to the search. The addition of these key terms had not been considered in the initial search strategy, and as a result of including them, the database returns increased significantly. The revised search was conducted in CINAHL Complete and Indigenous and databases. This process and search development is outlined in Appendix 1: Initial Search strategy and development. In order to get the most comprehensive contemporary picture of the resilience research, we limited our search 30 years.

The revised search strategy, including all identified keywords and index terms, was adapted for each included information source. The revised search using all newly identified keywords and index terms was then undertaken across CINAHL Complete, Medline, Web of Science, PsycInfo, PubPsych, and ProQuest databases. GREY literature was identified through searches of the National Health Medical Research Council, Australian Policy Online, Australian Institute of Health and Welfare, Informit Australian Indigenous; HealthInforNet, and Primary Health Care Research and Information Service. A further search was undertaken of Google Scholar to review and find any additional sources. Thirdly, the reference list of identified reports and articles were searched for additional sources. The keywords and subject headings used to search these databases are listed in Table 2, Search Terms.

**Table 2:** Search terms.

**Search terms**
indigenous OR native OR aborigin^*^ OR “Pacific Islander^*^” OR “Torres Strait Islander^*^” OR “First Nation^*^”
AND Australia OR Australian OR Australians
AND Resilien^*^ OR “mental health” OR Wellness OR “well-being” OR well-being OR strengths OR psychosocial OR “Protective factor^*^” OR “coping behavior^*^” OR coping skill^*^ OR growth OR Emotion^*^ OR Value
AND Conceptual^*^ OR perspective^*^ OR “world view” OR worldview OR narrative^*^ OR definition OR framework OR measure^*^ OR indicate^*^ OR meaning^*^ OR understanding OR perception^*^ or notion^*^ or attitude^*^ or knowledge OR belief OR Culture^*^ OR Kinship OR country OR land OR Dream^*^

### Study Selection and Outcome

Following the search, all identified citations were collated and uploaded into EndNote X9 (2020) version and duplicates removed. Titles and abstracts were screened by two independent reviewers, one Indigenous (RS) and one non-indigenous (JD), for assessment against the inclusion criteria for the review. Potentially relevant studies were retrieved in full and their citation details imported into the Joanna Briggs Institute System for the Unified Management, Assessment and Review of Information (JBI SUMARI) (Joanna Briggs Institute, Adelaide, Australia) (14). Full text screening of articles that passed title and abstract screening was carried out independently by the reviewers (RS/JD) in line with the inclusion and exclusion criteria. Reasons for exclusion of full text studies that did not meet the inclusion criteria were recorded and reported in the JBI System. Any disagreements that arose between the reviewers were resolved through discussion, or with a third reviewer (DJ).

**Figure 1:**
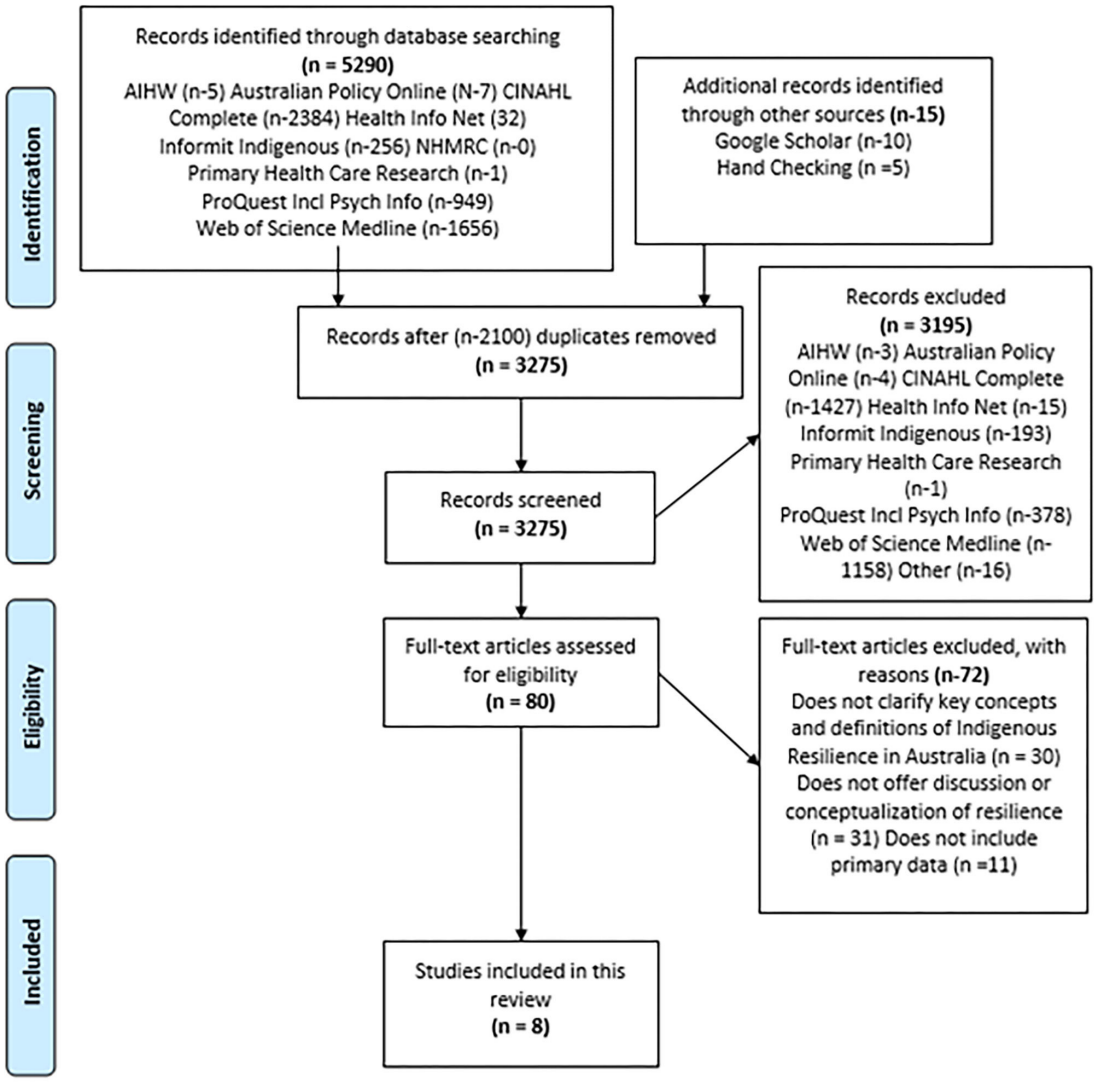
Prisma flow chart.

The search of the databases yielded 5,290 citations. An additional 15 citations were identified through Google Scholar and hand searching of references included in the full text screening process. A total of 2,100 duplicates were removed resulting in 3,275 citations for abstract and title screen and 3,195 were excluded. This was due to the domination of Western ideas with a concomitant marginalization of Indigenous views possibly reflecting publication bias. As the study was based on a strength-based approach, we were able to exclude a high number of studies that focussed on risk. Full text screening resulted in an additional 72 articles being excluded. The flow chart detailing this process is included in Figure 1. Prisma flow chart. The results of the search are reported in full in the final scoping review and presented in a Preferred Reporting Items for Systematic Reviews and Meta-analyses (PRISMA-P) flow diagram (15). A total of 8 studies were finally included in the current review. Included Studies with Characteristics and Key concepts are included in Table 3.

**Table 3:** Included studies with characteristics and key concepts.

**References**	**Title**	**Research Q/Aim**	**Study type**	**Effectiveness or acceptability**	**Geographical setting**	**Region urban, rural remote**	**Population**	**Context/Focus**	**Key concepts**
Dobia et al. (16)	Aboriginal Girls Circle: Enhancing Connectedness and Promoting Resilience for Aboriginal Girls: Final Pilot Report	1) To determine the effects of the Aboriginal Girls Circle on resilience, connectedness, self-concept and cultural identity. 2). To develop a culturally appropriate tools to measuring the constructs	Mixed methods Qualitative focus groups, interviews and observation Quantitative tool Aboriginal Elders, educators, community mentors	Peer reviewed	Dubbo, NSW	A Rural community in NSW	16 Aboriginal students in Aboriginal Girls Circle. 14 male and 11 female Aboriginal students were surveyed. In addition, 3 male and 13 female non- Aboriginal students were surveyed	The Aboriginal Girls' Circle is aims to increase social connection, participation and self-confidence amongst Aboriginal girls attending secondary schools	Supportive nurturing environment Program promotes inclusion, respect and skill to foster resilience An aim of the study was to understand how resilience is best conceptualized Indigenous Australian settings. It sought to identify commonalities and differences between existing formulations of resilience and the ways in which Aboriginal girls learn to build a sense of strength, confidence and well-being
Gale and Bolzan (17)	Social resilience: challenging neo-colonial thinking and practices around “risk”	Young men's perspective of resilience	Qualitative Interviews Did not apply measures of resilience	Peer reviewed	NE NSW	A rural community in Northern NSW	20 Young Indigenous Men	Developed and participated in a project PAWS-UP over 18 months	Focus on the themes and indicators central to social resilience highlighted by Indigenous young men
Gee (18)	Resilience and recovery from trauma among Aboriginal help seeking clients in an urban Aboriginal community-controlled organization	The development of a 60-item Aboriginal Resilience and Recovery Questionnaire Measures of historical loss and other risks	Mixed methods, in-depth interviews and structured questionnaire Developed 60 Item Aboriginal Resilience and Recovery Questionnaire (ARRQ)	Ph.D. Thesis	Melbourne, Victoria	Urban	Focus groups with *N* = ? Aboriginal health professionals 81 Aboriginal clients from the Family Counseling Services of the Victorian Aboriginal Health Service	Participant findings represent unique socio-historical and cultural resilience-based factors that are particularly salient for Aboriginal Victorians	Identified individual, interpersonal structural factors associated with resilience Developed a 60-item Aboriginal Resilience and Recovery Questionnaire with components that represent personal strengths and relational-cultural strengths The findings represent unique socio-historical and cultural resilience-based factors
Kickett (19)	Examination of how a culturally-appropriate definition of resilience affects the physical and mental health of Aboriginal people	The study aims to define resilience from an Aboriginal perspective	Qualitative Interviews Review of Indigenous literature of survival and success Did not apply measures of resilience	Ph.D. Thesis	WA	Perth	Aboriginal Elders and successful Aboriginal adults	Collected stories of survival and resilience over time Included a reflection of own lived experience growing up in WA through the assimilation era	Defined resilience as: • The ability to have a connection and belonging to one's land, family and culture, therefore an identity • Resilience that allows the pain and suffering caused from adversities to heal. • Having a dreaming, where the past is brought to the present and the present and the past are taken into the future • A strong spirit that confronts and conquers racism and oppression, strengthening the spirit • The ability not just to survive but to thrive in today's dominant culture
McLennan (20)	Family and community resilience in an Australian Indigenous community	Investigate the presence and complexity of resilience within an Indigenous Australian community, its relationship to well-being, and the implications an	A qualitative study with ethnographic and phenomenological design, utilizing semi structured, in-depth interviews and two focus groups with Yaegl Indigenous community members, between 2006 and 2010	Peer reviewed Research was part of a Ph.D. candidature	NE NSW	Rural The Yaegl Indigenous community in Northern NSW	15 In-depth interviews and 2 focus groups (24 Males−4 not Indigenous)	PAWS-UP Program promoting sense of belonging	Of particular significance is the importance participants placed on relationships for individual and collective strength and functioning. These relationships key to mediate risk and adversity, and foster community well-being. Existing and potential strengths and resources of a
		improved understanding of resilience may have for Indigenous health	Did not apply measures of resilience						community need to be recognized and valued in health and mental health service initiatives, as tools in preventing risk, strengthening recovery from ill-health or adversity, and enhancing well-being
Sivak et al. (21)	“Language Breathes Life” -Barngarla Community Perspectives on the Well-being Impacts of Reclaiming a Dormant Australian Aboriginal Language	Explore the contribution of language reclamation on well-being	Qualitative semi structured interviews Did not apply measures of resilience	Peer reviewed	Eyre Peninsular, SA	Rural community	16 Barngarla community members	Engagement with two of the three groups involved in pilot language reclamation project with a linguist	Confirms the contribution of language reclamation on improvements in mental health and Social Emotional Well-being, including strengthening resilience
Young et al. (22)	Perspectives on childhood resilience among the Aboriginal community: an interview study	To describe Aboriginal perspectives on the outcomes and origins of resilience among Aboriginal children	Qualitative Face to Face Interviews	Peer reviewed Very Strong Consolidated criteria for reporting qualitative research (COREQ) to inform study's design and reporting	NSW	Two urban and one regional Aboriginal Community Controlled Health Service in New South Wales	36 Aboriginal adults (15 health service professionals, 8 youth workers and 13 community members)	Study aims to inform programs to improve mental health outcomes for Aboriginal children HP's previously involved in the Study of Environment on Aboriginal Resilience and Child Health (SEARCH)	Resilience was broadly defined as ‘doing well despite problems children may face' Identified six themes: withstanding risk; adapting to adversity, positive social influences; instilling cultural identity; community safeguards; and personal empowerment
Young et al. (23)	The prevalence and protective factors for resilience in adolescent Aboriginal Australians living in urban areas: a cross-sectional study	To estimate the prevalence and determine protective factors for resilience in urban Aboriginal adolescents	Cross-sectional survey conducted between 2006 and 2012 Used Strengths and Difficulties Questionnaire	Peer reviewed	NSW	Conducted in partnership with four Aboriginal Community Controlled Health Services (ACCHS) in urban and large regional centers	120 of the 241 (50%) completed an adolescent survey (data for the remaining 121 were provided by their caregivers only)	The Study of Environment on Aboriginal Resilience and Child Health (SEARCH), a large-scale cohort study, to identify the determinants and trajectories of health in urban Aboriginal children and their caregivers	Based on previous SEARCH research, resilience was defined as normative social and emotional well-being. Resilience was defined/measured as having ‘low-risk' Strengths and Difficulties Questionnaire scores on the total difficulties (range: 0–40) or the prosocial scale (range: 0–10) Resilience was associated with nurturing family environments, social support and regular exercise

### Types of Sources

This scoping review considered both experimental and quasi-experimental study designs including randomized controlled trials, non-randomized controlled trials, before and after studies and interrupted time-series studies. While the search strategy included the terms experimental studies and randomized controlled trials, no papers of these types met inclusion criteria. In addition, analytical observational studies including prospective and retrospective cohort studies, case-control studies and analytical cross-sectional studies were considered for inclusion. Only one (23) was identified. This review also considered descriptive observational study designs including case series, individual case reports and descriptive cross-sectional studies for inclusion. Qualitative studies were considered that focused on qualitative data including designs such as phenomenology, ethnography, grounded theory, qualitative description, participatory action research, feminist research and decolonising research. Seven were included (16–22). None of the systematic reviews, texts or opinion articles considered met the inclusion criteria. Studies published in English since 1990 to ensure a comprehensive analysis of all pertinent literature were included.

### Data Extraction

Two PhD studies and six peer reviewed qualitative studies (two mixed methods) were included in the data analysis. Data extracted from the full texts of the studies occurred in two phases. The first extraction (RS and JD and DJ) focused on titles, meeting inclusion criteria, ideas, definitions of resilience and comments. The second extraction (RW) included the following: author, year; research question/aim; study design; study quality/effectiveness/acceptability; geographical setting; urban; rural remote); population (including sample size); context or intervention focus; and key concepts/outcomes reported.

Given the heterogeneity of study topics and populations an additional compilation of data was conducted to capture key concepts discussed across the eight studies encompassing individual, collective/community/social and environmental/structural resilience (see Table 4).

**Table 4:** Protective factors of aboriginal resilience.

**References**	**Social/Collective—Family/Community/Cultural connection**					**Individual Attributes/Skills**		**System/Structural/Environment**	
Dobia et al. (16)	Participation within the community	Stable supportive families home life provides for positive and meaningful community engagement	Cultural connection to community with reciprocal engagement, Being involved with community and Elders	Dimensions of cultural identity and pride, i.e., cultural events, learning cultural stories and protocols	Support from authentic role models Community support strongly associated with family and land	Positive self-image Self-confidence Problem solving Self-efficacy Self-awareness Empathy Co-operation	Education pathways Goals/aspirations	Positive participation within the school School engagement	Greater opportunities for educational attainment to overcome racism Understand Indigenous grief; be more positive and supportive
Gale and Bolzan (17)	Belonging and having a responsive community/civic connectedness	Working together to address tasks through kinship and friendship ties	Drawing on Indigenous approaches to knowledge and wisdom	Collective sense of self-determinationnot only about individual autonomyResponsibility to the land	Respect and Reciprocity – Having something to offer that is valued and accepted as worthwhile by ‘the other'	Being authors of own solutions,developing skills in organizing, communicating, negotiation and decision-making	Having agency to carry out those solutions; having hope for a future and creating new opportunities	Not being labeled; given respect as a group and having safety	Need to de-colonize the field of social welfare
Gee (18)	Interconnectedness of Individual, interpersonal structural factors Individual and family protective factors strong family support and cohesion	Experiencing belonging and being rooted to land, family, culture, and spirit as a source of resilience	Connecting with culture community, building Cultural pride Opportunities for healing	Cultural attachment Participation in traditional activities Connections to land and language	Sense of pride in cultural identity and counseling support	Feeling culturally safe. Sense of self esteem Sense of control. Thinking healthy. Making positive choices relationship to mainstream	Conquering social adversities such as racism and oppression	Provision of information about chronic disease and mental health Adequate access to and provision of services and support, access to Aboriginal community-controlled and other community services, and receiving continuity of care and service support Supporting men's and women's cultural support groups, counseling Breaking down institutionalized racism and negative stereotypes in mainstream	
Kickett (19)	Identity, culture, spirituality, family land, environment, sense of belonging and connectedness	Parental strength and encouragement	Cultural values, practices and beliefs have a profound impact on individual, family and community resilience	Access and management of traditional lands participation in cultural practices and storytelling for transmission of cultural knowledge	Having a sense of hope and an ability to adapt, cope and learn Sense of spirituality linking beliefs in being strong and dealing with grief and loss and healing	Having a positive attitude linked to self-esteem Adapting, managing in two worlds; coping with adversities and having a sense of humor	Setting Goals Understanding knowledge is power, learning to cope with injustice and inequity, discrimination and racism	Need for programs and health professionals to recognize Indigenous perspectives of resilience The need to support an Aboriginal Framework of Resilience which fosters healing, and strengthening families and connectedness to culture, family, community land spirituality	
McLennan (20)	Nurturing environment Community, school	Family Sharing and Affection with community	Culture and Kinship Connectedness Knowledge of cultural heritage	Leadership Strengthening relationships	Role Models i.e., Elders grandparents extended family and teachers	Strength determination stubbornness Keep going	Increase youth participation	Increase access to health information Health and mental health services need recognize and support Aboriginal community strengths and resources Supporting parents	
Sivak et al. (21)	Connection to community	Connection to family and kin	Connection to country, spirituality Ancestors Sense of identity and Pride	Connections to culture and heritage Acknowledging past generations and recognizing of what they endured	Mending of community through language reclamation	Self-confidence Self esteem (linked to mind and emotions) Cultural pride at an individual level	Personal empowerment Increasing personal growth, empowerment	Language reclamation	
Young et al. (22)	Positive social influences	Healthy behaviors and relationship with family and community	Cultural Identity	Children's connection to culture fosters a sense of belonging and pride in their ancestry	Shared responsibility Positive role models	Personal Empowerment		Community safeguardsproviding enriching opportunities	Strategic sustainable services, holistic support
Young et al. (23)	Resilience was associated with nurturing family environments, social support and regular exercise	Nurturing family	Cultural identity Cultural knowledge	Access to traditional lands and its management Participation in cultural practices Storytelling for transmission of cultural knowledge	Role models Social groups	Self-belief Self-esteem To set goals	Positive pathways Education	Access to services	Holistic programs

### Quality Appraisal

As outlined we used a decolonizing approach in this review. Throughout this process, we were conscious of the potential impact of using a quality appraisal to exclude sources of information that do not meet Western research standards. Of particular concern were questions or judgements the tools ask authors to cast on research, particularly as they relate to the appropriateness, justification for and value of the research (24). As previously outlined we extracted information on quality/effectiveness/acceptability which ensured studies were peer reviewed and had appropriate ethical approval to carry out the research. No formal quality appraisal tool was used, nor is one required for a scoping review.

### Interpretation of the Data

Given we were determined to present a different view to the current Western dominated understandings of Indigenous resilience, it was important our analysis of the evidence incorporated a decolonizing approach. To do this, we worked closely as a team but also took several iterations of the analyses back to the Aboriginal Research Cultural Advisory Groups for their input and discussion. In this way, reflexivity through collective dialogue was pivotal to the analytic process (6). In addition to incorporating a reflective decolonizing collective dialogue to reframe our exploration of the concept of resilience, we explored the notion that the influence of dominant discourses could impede the Indigenous definition of resilience causing Indigenous voices to go unheard if not given a safe place to be shared (25). By acknowledging this tension, we transformed our definition through creating space to understand resilience and interpret notions of resilience led by Indigenous ontologies and epistemologies. By incorporating this approach to analysis, we were able to adopt a critically reflexive lens to interrogate the extent to which adherence to Western constructions and measures of resilience may have influenced interpretation of ours and previous findings. It also provided a means for non-indigenous authors to examine their assumptions regarding the notions of resilience and adversity and ensure they privileged the voices of both the Indigenous authors and participants within the included studies.

## Results

All eight eligible studies in this review discussed Indigenous perspectives of resilience. They extended Western conceptions, going beyond the ability of individuals to cope in the face of adversity. For example, McLennan (20) describes how individual and collective relations enhance Indigenous community sense of well-being and ameliorate risk and adversity. Also evident in several studies (16, 18, 19) is the way adversity encompasses the experience of coping with the historical and contemporary impacts of colonization, including issues of racism and structural inequalities. Dobia et al. (16) and Gee (18) also suggest the high frequency of grief and loss experienced by Aboriginal people contributes to the significant burden of cumulative and transgenerational trauma. While Gee (18) explores these issues from a clinical and therapeutic perspective, Dobia et al. (16) highlight the importance of education to overcome racist stereotypes and the need for schools to promote understanding and manage grief from an Aboriginal perspective to become more positive and supportive environments.

Overall the studies obtained Indigenous perspectives on resilience from a range of different Indigenous populations in four states in Australia: New South Wales, Victoria, South Australia, and Western Australia, representing gender diversity and different age groups (see Table 3). Several studies also identified a range of Indigenous perspectives related to both adversity and the strategies or interventions required to strengthen resilience. An analysis of the results of these studies show that while there are some striking commonalities among them, there are also some differences reflecting the needs of different population groups and circumstances or contexts in which the studies were conducted as well as their different aims. Three studies applied tools to measure resilience. In one study (23) mainstream tools were used to measure resilience which have not been validated in Indigenous settings, in two studies (16, 18) the researchers worked with Indigenous communities to develop the measuring tools.

The main themes discussed in most of the studies which address the aims of the scoping review include: the definitions and frameworks of resilience; characteristics contributing to individual and collective/social resilience; measuring resilience; and, implications and strategies for policies and practice (service provider and clinical) to promote resilience. These themes are discussed in detail below.

### Theme 1: Indigenous Concepts and Definitions of Resilience

Most of the studies linked resilience to well-being or mental health. The findings from McLennan (20) indicate that resilience is multi-layered with multiple family and community sources of protection, support and resources necessary to foster strength and well-being (resilience) in response to adversity and hardship. Key family and community protective sources include connectedness, sharing and affection, role models and leadership. Relationships are key to the sense of well-being of and within the community.

Gale and Bolzan (17) focussed on social resilience and highlighted the need to acknowledge the role of historical, economic and political factors in influencing individual, family, and community resilience. They discuss how the ongoing impact of neo-colonial practices fails to acknowledge Indigenous strengths or the adverse impacts of social determinants, focusing instead on Indigenous People “as being at high risk and requiring intensive intervention and governance” (p. 1). The authors argue that most studies of Aboriginal people are risk oriented, focusing on individual and family failure; “problematic schooling,” unemployment of parents and absent fathers without regard to social circumstances and processes that contribute to such specific risk factors. They identified several themes related to social resilience emerging from their interviews with young men. These included: being authors of their own solutions, having agency to carry out those solutions; not being problematized or labeled negatively; given respect as a group and having safety; civic connectedness, belonging and having a responsive community; and having hope for a future—flourishing not just surviving.

Similarly, in contrast to deficit constructions of resilience, drawing on an analysis of interviews with successful Aboriginal Elders and successful Aboriginal people in Western Australia, Aboriginal scholar and educator, Marion Kickett (19) defined resilience as:

*The ability to have a connection and belonging to one's land, family and culture, therefore an identity. Allowing pain and suffering caused from adversities to heal. Having a dreaming, where the past is brought to the present and the present and the past are taken into the future. A strong spirit that confronts and conquers racism and oppression, strengthening the spirit. The ability not just to survive but to thrive in today's dominant culture*. (2011, pii)

This definition was reinforced by Gee's (18) interview findings in his extensive study with Aboriginal health practitioners and Aboriginal clients seeking counseling in an Aboriginal community-controlled health service in Victoria. Gee (18) suggests that resilience processes and outcomes are generally seen as the interplay between risk and protective factors, where the protective factors modify the risks. Gee also makes the point that a review of literature focused on Aboriginal resilience found Aboriginal people identified similar individual and family level protective factors as in non-Aboriginal populations (e.g., the protective effects of self-esteem, mastery, parental support, and family cohesion). However, citing Kickett's (19) research, Gee notes that an important difference was the evidence for protective effects of cultural constructions of resilience across a range of different social and emotional well-being outcomes. Indigenous specific factors that emerged in the research conducted with Indigenous Peoples, include sense of pride in cultural identity, cultural attachment, participation in traditional activities, and connections to land and language [Kickett (19), p. 139]. Drawing on Kickett's definition, Gee (18) argues that in addition to recognizing the ability to overcome adversity, this Aboriginal understanding of resilience highlights the importance of experiencing belonging and being rooted in the land, family, culture, and spirit as a source of resilience and the need to conquer social adversities such as racism and oppression. As Table 4 confirms many of the studies in our review found similar results confirming the importance of individual factors as well as the distinct role of collective, cultural and environmental elements influencing resilience.

Citing Aboriginal perspectives with respect to childhood resilience, Young et al. (22) defined resilience as the ability to endure adversity with minimal disruption to normal development and social functioning and the strength to choose positive behaviors in the face of challenging circumstances. Drawing on their previous qualitative research findings with Aboriginal families participating in the Study of Environment on Aboriginal Resilience and Child Health (SEARCH) and their quantitative survey, Young et al. (23) suggested education and supportive familial and social environments as important for developing resilience in children.

### Theme 2: Frameworks of Resilience

A socioecological model of resilience based on Bronfenbrenner's (26) theory, which emphasizes the importance of connectedness, relationality and a supportive environment in supporting individual development, was referred to in most of the studies reviewed. Dobia et al. (16) adopt a socioecological model of resilience, citing connectedness and cultural identity as positive aspects of resilience highlighted by Aboriginal community members. Dobia et al. (16), Gale and Bolzan (17), Gee (18), and McLennan (20) all draw on Ungar's critique of Western conceptions of resilience which fail to recognize how contextual and cultural processes are relevant to resilience of marginalized youth (27, 28). Ungar et al. describe resilience as “*both an individual's capacity to navigate to health resources and a condition of the individual's family, community, and culture to provide these resources in culturally meaningful ways”* [(29), p. 10] Drawing on a widely representative range of Indigenous participant perspectives, several of the studies (16–18, 20) affirm this definition, identifying the need to strengthen the capacity of communities and services to facilitate a positive environment to strengthen individual resilience in response to risk and adversity. A study of Indigenous young men by Gale and Bozan (17) highlights the importance of civic connectedness and responsive communities to promote resilience. These authors examined the importance of connectedness for participants in relation to family, school, community, and culture and identified themes supporting resilience based on the literature and interview findings. These themes include: *School connectedness* to mitigate behavioral problems and mental health problems and enhance student achievement.

Similar to studies of Indigenous perspectives of resilience in Canada (30, 31), several studies (18, 19, 22) identified socialization and sense of cultural connectedness as key protective factors in developing an individual's resilience. A positive and strong sense of cultural identity; knowledge of traditional cultural beliefs and values; participation in cultural activities and practices, and engagement in cultural gatherings to promote individual and collective resilience were identified in the studies by Gee (18); Kickett (10); McLennan (20); and Young et al. (22, 23). Focusing on social resilience, the study by Gale and Bolzan (17) stressed the importance of cultural identity and connection as major protective factors promoting resilience. Kickett (19) and Young et al. (23) point out that having access to traditional lands and its management and participation in cultural practices and story telling supporting the transmission of cultural knowledge promote resilience, physical health, and social and emotional well-being (SEWB).

McLennan's study sought “to investigate the presence and complexity of resilience within an Indigenous Australian community, and its relationship to well-being” [(20), p. 2] to improve and inform new directions for health promotion and service provision. McLennan suggested that the concept of resilience was implicit in participants use of terms and phrases, including “strength,” “determination,” and “stubbornness,” and reporting factors that helped them feel supported and to “keep going.”

Drawing on the perspectives in these studies it is evident Indigenous communities experience distinctive adversities and risk as well as protective factors. Sivak et al. (21) did not focus specifically on resilience, rather the authors examined whether participating in language reclamation contributes positively to and aligns with the SEWB framework proposed by Gee (18). Key themes included: connection to spirituality and ancestors; Country; culture; community; family and kinship; mind and emotions; and impacts upon identity and cultural pride at an individual level. Whilst not stated, there is an implicit collective nature within Sivak et al.'s (21) key themes.

Community participants described the power of connections to culture and heritage and to the strength and resilience of past generations and what they went through and endured “*to still be alive today is so powerful. So there's a lot of strength that comes through so many generations.”* (p. 8). With respect to the theme of connection to mind and emotions participants spoke of “increasing strength, resilience, personal growth, empowerment, and mending of community” (p.11). A sub theme of the overarching concept of social and emotional well-being involved intergenerational knowledge transfer; engaging all age groups in sharing language, dreams and aspirations for the community for the future and promoting a sense of belonging.

Family and community gatherings were also identified as a way “*for people to remind one another of their resilience and ongoing connection to a strong family, community, lineage and Country. The feelings of belonging fostered through the language-based gatherings allowed participants to help the group, and the generations to come”* (p.13). A second sub theme examining the impact of language reclamation on well-being domains include: happiness and excitement; recognition; resilience; optimism and positivity; motivation; empowerment and self-esteem; self-confidence and personal growth; and pride.

McLennan (20) categorized protective factors as: individual; family; and community-based domains; acknowledging that some may develop from the interaction between these three domains, with individual character traits and coping influenced by parenting and role models. This study focused on community and family based protective factors in contributing to the development of resilience and support of well-being within communities.

In discussing Aboriginal community and health provider perspectives with respect to developing resilience in Aboriginal young people, Young et al. (22) identified six themes encompassing both internal and external factors. These were: withstanding risk; adapting to adversity; positive social influences; instilling cultural identity; community safeguards; and personal empowerment.

### Theme 3 Key Characteristics Related to Indigenous Resilience

Protective factors of Aboriginal resilience were identified through the literature (see Table 4) and involved individual attributes and skills as well as social/collective, family/community and systemic/structural factors.

#### Individual Influences

##### Internal Coping

Participants in the study by Young et al. (22) focused on individual attributes suggesting the ability to withstand risk (displaying normative development, possessing inner fortitude) and adapting to adversity (necessary endurance, masking inner vulnerabilities); contribute to resilience. Further, participants believed that “children who experienced adversity, but who were able to show empathy, take pride in their appearance, show respect for themselves and others, maintain prosocial relationships, regularly attend school and value education” were resilient [(22) p. 406].

##### Self-Concept

Dobia et al. (16) revealed the importance of significant associations between internal resilience factors (self-concept and greater levels of self-esteem) and environmental resilience and meaningful home and community participation, prosocial peers, and home support as positively influencing well-being. General self-esteem was also associated with internal resilience, and was the only self-concept factor associated with internal resiliency, they highlight the need for “a positive sense of self in the face of racist cultural stereotypes” (p. 11).

##### Personal Empowerment

In Young et al.'s (22) study, participants emphasized the importance of establishing positive pathways to hope and resilience for children for them to develop self-respect and make positive decision-making choices. Several study participants believed that children with a sense of self-esteem and self-efficacy, able to set, pursue, and achieve their goals are more likely to have the resilience to persevere in the face of adversity.

##### Interpersonal Qualities

In Gale and Bolzan's (17) study, participants identified a range of individual qualities and interpersonal skills that contribute to young people's resilience as being responsible to each other, communicating and negotiating decisions, setting rules around groups, and strengthening kinship and friendship ties. They also emphasized their commitment to a collective framework and *their responsibility to the group and the land* [(17), pp. 13–14 emphasis added]. This study confirmed the interplay between individual and social/cultural protective factors that contribute to resilience.

#### Social Influences

##### Secure Family Environment

Young et al. (22) found that while some participants feel resilience is an innate quality, many believe resilience can be learned and nurtured within supportive family and community interactions. The study findings in Kickett (18, 19, 22, 23) suggest resilience is fostered within secure family environments that promote positive role models, healthy behaviors and relationships. Dobia et al. (16) also highlight the importance of family relations and McLennan (20) suggests the importance of affection and sharing within families as supporting resilience in young people.

##### Community Safeguards

Several studies stressed the need for communities to provide the foundations for building and maintaining resilience. For instance, Young et al. (22) highlighted the need for communities to offer strategic sustainable services, holistic support, shared responsibility, and providing enriching opportunities.

##### Role Modeling and Leadership

Aboriginal participants in all studies discussed the importance of positive role modeling to provide individual guidance and well-being within the community. Many participants described having role models including parents, uncles, aunts, Elders and schoolteachers as a source of inspiration, and saw people exhibiting leadership within the community as a motivating factor.

#### Cultural/Community Influences

##### Instilling Cultural Identity

Young et al. (22) highlighted the need to invest in Aboriginal knowledge to build a strong cultural self-concept. Participants believed children's connection to culture fosters a sense of belonging and pride in their ancestry, generating strength during challenging times. Having a clear, strong and positive self-concept as an Aboriginal person makes people more resilient to the discrimination and negative stereotyping experienced in White society. A strong sense of cultural identity and safe, stable and supportive family environments were thought to promote resilient behaviors.

##### Strong Community Connections

An analysis of Aboriginal community and Aboriginal teachers' perspectives confirmed the importance of strong community connections in building bonds between young women and community resilience (16). The findings reinforced the importance of connecting local Elders and community members with young girls to build their sense of cultural identity and self-esteem, and to strengthen their connections within the community. All of the students nominated cultural camps; time with Elders; meeting new people; circle activities, and the ability to connect to their culture and other Aboriginal girls as important activities which strengthen their sense of cultural identity and connection.

##### Family and Community Connectedness

Making a strong link between well-being and resilience, McLennan (20) noted that the interdependent nature of individual well-being with family and community well-being was discussed repeatedly throughout the interviews and focus groups. Togetherness experienced at times of funerals, the occasional cultural gatherings and the more regular BBQ get-togethers were noted as important elements. According to the findings by Young et al. (23), it is likely that increased school attendance strengthens resilience through regular socialization with peers. Families that encourage adolescents to attend school are likely associated with other factors including nurturing parenting and family cohesion that build resilience. The authors stress the importance of a cohesive family environment and positive parenting behaviors in promoting good mental health which they link with resilience. Dobia et al. (16) suggested resilience was strongly related to a number of cultural identity dimensions, such as taking part in cultural events, learning cultural stories and protocols, being involved with community and Elders and taking pride in one's culture. These dimensions also yielded significant correlations with community support which was also strongly associated with family and land.

Importantly drawing on Aboriginal perspectives Gee (18); Kickett (19), McLennan (20), Young et al. (22) highlight the critical role of connection to culture as an important determinant of health and resilience. While Young et al. (23) claim they found no significant relationship between cultural knowledge and resilience and point to other studies that also had inconsistent results with the above studies, what is evident is that none of the studies used Indigenous measures of resilience, with most adopting the Strengths and Difficulties Questionnaire which, while widely used in Indigenous studies, has not been validated for use with Indigenous participants (32).

##### Affection and Sharing

Many of the participants in the McLennan (20) study emphasized the importance of affection and sharing among community members to support each to other overcome adversity, particularly in the form of grief. Elders spoke of sharing resources including food and income in times of hardship. Others highlighted the community's ability to care for one another as important.

##### Social Connectedness

While several studies confirm the important role of cultural connectedness, the study by Gale and Bolzan (17) identifies the importance of *cross-cultural connections* as contributing to social resilience. For instance, the PAWS-UP project meant that young people were involved in activities that increased their connectedness with the wider community. These civic connections and the resultant community responsiveness were pivotal to the young people's transformation and enhanced social resilience. Gale and Bolzan (17) show that social resilience involves the communities ‘*capacity to be responsive to Indigenous Peoples' active participation in its formation and direction*' (p. 23). Social resilience is both a process and outcome resulting in an enhanced sense of agency, changes in local community perceptions and power relations, and increased civic connectedness and community responsiveness between Indigenous and non-Indigenous groups. This highlights the importance of programs that engage Indigenous young people in *whole community* activities that transform perceptions and foster social resilience.

### Theme 4: Tools for Measuring Resilience

Building on the emphasis of connectedness identified in the qualitative aspect of their study, Dobia et al. (16) measured connectedness using relevant items from the California Healthy Kids Survey (CHKS) using the Resilience and Youth Development Module (RYDM) environmental resiliency scale. This measure assesses both internal resilience (personal strengths and communication skills), and environmental and social factors that contribute to resilience which enables analysis of the relationships between environmental risk and protective factors and internal strengths. It measures of the extent to which individuals feel connected and supported at home, school and in the community. Considering the views of Aboriginal participants and insights from Aboriginal Australian research literature a cultural identity measure was developed with nine factors identified as being important components of students' sense of their Aboriginal identity (with an additional factor assessing personal experiences of racism).

The development of this measure incorporated Aboriginal voice and included: *Cultural Pride, Cultural Learning, Cultural Protocols, Cultural Elders, Cultural Family, Connection to Country, Cultural Mob, Cultural Events, Cultural Community Support and one measure of experiences of racism* [(16), p. 21]. These nine dimensions of relatedness were assessed across school, community, family, and peers to measure resilience. They drew on evidence which confirmed that a strong sense of Aboriginal culture and sense of belonging, connectedness and self-worth supports positive mental health and well-being (which is linked to resilience). Young et al. (22) suggest the ability to be resilient was identified as a “necessary ability” for Aboriginal adolescents to maintain good mental health. This was supported by the qualitative interviews with Aboriginal Girls Circle (AGC) participants, Aboriginal staff and community members. They also identified that a positive sense of cultural identity can be a source of resilience against the impacts of racism [(16), p. 20].

Their findings demonstrate strong support for the association between a positive sense of cultural identity and the resilience and well-being of Aboriginal youth (16). The study also emphasized the importance of young people learning about Aboriginal culture to support positive development, confidence and strength and direct interactions with local Elders and community members to build cultural identity and self-esteem and strengthen connections within the community.

Gee (18) used structured interviews to measure historical loss, stress, depression, drug and alcohol use, empowerment, resilience as coping with stress, and personal, relational-cultural and global strengths. The findings informed the research and design of an Aboriginal Resilience and Recovery Questionnaire (ARRQ) (Study One) which was then applied by Gee in Study Two. It included two sub-scales, personal strengths and relational-cultural strengths, using this measure along with measuring the cultural idioms of distress included in the Aboriginal Australian Version of the Harvard Trauma Questionnaire (AAVHTQ), to interview 81 Aboriginal clients from the Family Counseling Services. Gee (18) revealed high levels of trauma among clients. He found that two generations of child removal, historical loss, experiences of racism, limited living expenses were associated with greater trauma symptoms severity. Conversely, clients exhibiting personal and relational-cultural strengths, and global strengths, were associated with lower trauma and depression symptom severity, and less drug and alcohol use. Furthermore, participants who had participated in healing from past trauma reported positive emotions, strong relationships (attachment), feeling safe, resilience as coping with stress and personal and relational-cultural strengths, and global strengths. Importantly, Gee (18) also found that the ARRQ shows promise as a measure that can be used by Aboriginal counseling services across Australia to better assess the extent to which its therapeutic practices and programs support Aboriginal help-seeking clients in increasing their strengths and resources, and experiencing healing and trauma recovery outcomes.

### Theme 5: Strategies to Strengthen Protective Factors Within Individuals, Families, and Communities

An analysis of literature by McLennan (20) confirm that participation in family and community well-being programs and men's group activities improves individual empowerment, sense of self-worth, resilience and problem-solving ability, and capacity to strengthen their families and communities. In addition, McLennan identified several strategies to strengthen community including: increasing access to medical information and services; enhancing community cohesion, by encouraging closeness and increasing community gatherings and participation; increasing youth participation, respect and knowledge of their cultural heritage and kinship ties, through community activities and education; for families assisting parenting and financial management skills through support and education; and improving cultural identity and pride within the community, by passing on specific cultural knowledge from older community members and Elders (2015, p. 5). McLennan also stressed the importance of role models.

The study by Dobia et al. (16) using survey measures developed with Aboriginal people and drawing on Aboriginal literature, provides a nuanced understanding of Aboriginal mental health and well-being and the value of programs such as AGC which include a range of components to support individual and collective relations that are likely to be effective in enhancing resilience among young people. Using the ARRQ, Gee (18) also confirmed that cultural practices were a predictor of empowerment, partially mediated by self-esteem. His second study indicated a range of important risk and protective factors that influence post trauma outcomes among Aboriginal clients attending the Family Counseling Services. While some factors are consistent with the post-traumatic stress disorder (PTSD) and Complex PTSD recovery literature, Gee's study revealed unique socio-historical and cultural-resilience based factors that influence Aboriginal client outcomes.

### Implications for Policy and Practice

Young et al. (22) suggest that the implications for public health policy and practice require more sustainable, Aboriginal-led programs to strengthen positive family dynamics, identify children at-risk and provide safeguards during periods of familial adversity. Several studies confirm the need for Aboriginal people to have improved access to culturally responsive health and social services and health information (18, 22). Gee's study provides important insights into the therapeutic value of cultural healing in addition to clinical treatment as a critical element when working with Indigenous People to support recovery from transgenerational and contemporary and cumulative trauma, grief and loss. Community level interventions promoted through the Aboriginal and Torres Strait Islander Healing Foundational found that over 10,000 Indigenous people participating in 21 Indigenous healing projects that supported cultural connection and reclamation reported positive outcomes across three key domains of well-being that strengthen resilience. Overall 92 per cent reported “Strengthened physical, emotional, social and spiritual well-being” (national outcome one), 95 per cent reported “*Strengthened connection to culture” (national outcome two), and 94 per cent reported “Strengthened pride in cultural identity”* (national outcome three) [Gilmour (33) p. 15]. The study by McLennan (20) found that broad ranging, interdependent protective factors were indicated within family and community systems, including supportive processes, community cohesion, love and support, role-modeling and leadership, affection and sharing, friendship, and culture. Their study highlights the need for greater research into the intersection between Indigenous community health and well-being and resilience, in order to build strengths-based models of health care and rehabilitation (or cultural reclamation) with and by Indigenous People. Utilizing, and building on, existing community strengths, capacities and resources is key to strengthening resilience. These resources need to be recognized and valued in health and mental health service initiatives, including their potential to be utilized as tools in preventing risk, strengthening recovery from health or adversity, and promoting well-being. The studies by Kickett (19) and Sivak et al. (21) confirm that the role of culture and language reclamation in supporting well-being is also critical. The results of the study by Young et al. (23) confirm the need for strategies to support Aboriginal families to have the ability and capacity to support and encourage their children's education to increase resilience in Aboriginal adolescents. They also highlight the need to provide social, scholastic support and physical/sporting programs for Aboriginal young people to foster greater self-efficacy and self-esteem that can lead to resilience. Their recommendations fall short on most of the studies that emphasize the need for programs that promote cultural connection, enhance cultural identity, links with Elders, ancestry, and connection with Country.

## Discussion

The aim of this scoping review was to incorporate Indigenous ways of knowing, being, and doing to produce authentic insights and more culturally inclusive understandings of resilience than has previously been achieved. This review allowed us to push to extend beyond the dominant, non-Indigenous definitions of resilience to better understand resilience from the perspective of Indigenous Peoples of Australia. Using a decolonizing approach, the literature review revealed a different narrative from our initial attempt at the review. If we had not persisted in our quest to truly understand resilience from an Indigenous perspective, we would have continued the same narrative to previous reviews that ignored the possibility of a different perspective on resilience. Using a decolonizing approach, we began to peel away the layers and reflect on ways in which we began to understand the multiplicity of elements within the concept of Indigenous resilience, depending on an individual's situation. Importantly, the results we gathered allowed our work to expand beyond the individual and their ability to cope with adversity to acknowledge wider viewpoints encompassing family, culture and community, that more closely reflect Indigenous perspectives and understandings of resilience (34).To articulate this perspective, Kickett (19) argues the notion of resilience must be made explicit, focusing on the individual, accessing land, family and culture and developing their identity, moving toward agency, determination and spirit, to not just survive, but thrive (19). Particularly, thriving in the face of adversity such as the impacts of colonialism and racism, to focus attention toward the importance of culture, strength and belonging.

The review supports previous findings that show the links between adversity and resilience and affirm the determination of communities (as a collective) that draw on their cultural knowledge and traditions to transform their situations in order to “thrive not just survive” (19). Importantly, in many of the review studies adversity was linked to the enduring legacies of colonization, continuous and cumulative transgenerational grief and loss, structural inequities, and racism and discrimination (16, 18, 19). The studies confirm that these external factors of adversity are unique to Aboriginal populations, as are the protective factors that entail strengthening connection to culture (including language reclamation), community, ancestry and land (including management and economic development) which contribute to individual and collective resilience. These findings go further than focusing on individual coping and personal development to promote resilience previously discussed in the literature. They suggest that Indigenous community resilience is strengthened through the collective experience of adversity, such as transgenerational grief and loss, and the resulting support structures and shared resources that are developed and maintained through cultural practices to “strengthen the bonds and mutual reciprocity” to participate in transformative strategies to address adversity. Several studies suggest that reframing resilience to embrace Indigenous perspectives has the potential to promote well-being in Indigenous communities across Australia.

## Limitations and Opportunities for Further Research

As with all research, this review has some limitations. A limitation of this review is that this review was limited to peer review, published articles and we acknowledge that Indigenous authors are under-represented in the literature, perhaps because of publication bias and the continued challenges faced by Indigenous researchers in Australia. Further, we acknowledge that Indigenous peoples have other forms of disseminating information including the spoken word, storytelling, art and other important knowledge sharing methods. We did not capture evidence disseminated in these ways.

Furthermore, in this paper, we attempted to draw together unifying concepts, however, we acknowledge that these may not reflect the needs of the extremely diverse Indigenous populations across Australia. We would therefore advise any future researchers who are focussed on resilience to engage in active dialogue with community to achieve an in-depth understanding of what Indigenous resilience means within a local context.

Gender was presented as a consideration within many of the included studies presented. This provides an opportunity for further research focussed on issues of gender and understanding and promoting Indigenous Resilience.

## Conclusion

This review confirms the critical value of adopting a decolonizing lens to examine Indigenous concepts of resilience in order to reveal understandings for policy, programs and practice, that were hidden under Western definitions and understandings of resilience. The studies analyzed in this review reveal both the distinctive colonial characteristics of adversity experienced by Indigenous people and the range of coping strategies and protective resources that support the development of resilience within different Indigenous communities in diverse sites across Australia. This review highlights that resources such as building on community strengths, capacities and resources is critical when strengthening resilience within Indigenous communities across Australia.

## Data Availability Statement

The original contributions presented in the study are included in the article/Supplementary Material, further inquiries can be directed to the corresponding author/s.

## Author's Note

The review involved the participation of local Aboriginal Research Cultural Advisory Groups who participated in the review, approved the analysis of the findings and collaborated on the design and writing of the paper.

## Author Contributions

KU, DJ, RM, and JD: initial conceptualization and design of the review. RS and JD: all literature searching, screening, and appraisal. RW: data analysis and results presentation. UNS, IA, MR, and CP: guidance and cultural advice throughout the review. KU, DJ, RM, JD, RW, and RS: writing, redesign, and presentation of the paper. All authors contributed to the article and approved the submitted version.

## Conflict of Interest

The authors declare that the research was conducted in the absence of any commercial or financial relationships that could be construed as a potential conflict of interest.
