# Supplementary material for: Indigenous Resilience in Australia: A Scoping Review Using a Reflective Decolonizing Collective Dialogue
Source: Front Public Health. 2021 Mar 31;9:630601. doi: 10.3389/fpubh.2021.630601 (PMC8044395; doi:10.3389/fpubh.2021.630601)
Supplement: Supplementary file 2 [file Data_Sheet_2.DOCX]

## Appendix 1: Initial Search strategy and development

| **Search Strategy** | **Results** |
| --- | --- |
| indigenous OR native OR aborigin* OR "Pacific Islander*" OR “Torres Strait Islander*” OR "First Nation*"  2. Australia OR Australian OR Australians  3. Resilien* OR "mental health" OR Wellness OR "well being" OR wellbeing OR  strengths OR psychosocial OR "Protective factor*" OR "coping behavio*" OR  coping skill*  4. Conceptual* OR perspective* OR "world view" OR worldview OR narrative* OR  definition OR framework OR measure* OR indicat* OR meaning* OR  understanding OR perception* or notion*  **Limits**: SU – Subject; Date 1990-2020; English Language | CINAHL Complete  RESULTS: **984.**  Search conducted on 7 June 2020  Indigenous Collection  RESULTS: **82**  Search conducted 26 June 2020 |
| Following review of subject terms on the returned articles, eleven additional key words were included in a revised search: *Culture, Kinship, Country, Dream*,Social, Growth, Emotion* ,Attitud*, Knowledge, Belief, Value** | Revision of terms |
| indigenous OR native OR aborigin* OR "Pacific Islander*" OR “Torres Strait Islander*” OR "First Nation*"  2. Australia OR Australian OR Australians  3. Resilien* OR "mental health" OR Wellness OR "well being" OR wellbeing OR strengths OR psychosocial OR "Protective factor*" OR "coping behavio*" OR coping skill* OR growth OR Emotion* OR Value  4. Conceptual* OR perspective* OR "world view" OR worldview OR narrative* OR definition OR framework OR measure* OR indicat* OR meaning* OR understanding OR perception* or notion* or attitude* or knowledge OR belief OR Cultur* OR Kinship OR country OR land OR Dream*  **Limits**: SU – Subject; Date 1990-2020; English Language | CINAHL Complete RESULTS: **2,384**  Searched conducted 26 June 2020  Indigenous Collection RESULTS: **256** conducted 26 June 2020 |
